# AFFECTIVE AND COGNITIVE CONSEQUENCES OF PERCEIVING DAILY EXPECTATIONS FOR ACTIVE AGING

**DOI:** 10.1093/geroni/igae098.1736

**Published:** 2024-12-31

**Authors:** Maria Wirth, Klaus Rothermund

**Affiliations:** Friedrich Schiller University Jena, Jena, Thuringen, Germany; Friedrich Schiller University Jena, Jena, Thuringen, Germany

## Abstract

Older individuals face increasing social expectations and demands to remain active and participate in society. Perceived expectations for active aging (PEAA) refer to older adults’ recognizing such expectations as being directed at them personally. Previous studies, investigating PEAA as a relatively stable trait-like construct, showed that most individuals can cope well with activation demands. However, some individuals might be distressed by them. A deeper understanding of PEAA and their consequences can be reached when both are assessed in everyday life. In a 14-day diary pilot study with 93 participants (Mage = 65.40, 50-84 years), we assessed daily PEAA in the physical, cognitive, social, and appearance domains and related them to daily positive and negative affect as well as subjective age. Complementing previous work, we showed that daily PEAA not only differed between but also within individuals and that these variations were related to affective experience and subjective age. In multilevel models controlling for daily hassles and uplifts, we showed that on days on which individuals perceived more expectations for active aging, they also reported more positive affect. Daily PEAA and daily negative affect were not related. Moreover, on days on which individuals reported more daily PEAA, they also reported feeling younger. These findings point to the importance of assessing and understanding PEAA within older individual’s daily context.

